# Pattern of congenital anomalies at birth and their correlations with maternal characteristics in the maternity teaching hospital, Erbil city, Iraq

**DOI:** 10.1186/s12884-018-2141-2

**Published:** 2018-12-18

**Authors:** Sozan K. Ameen, Shahla Kareem Alalaf, Nazar P. Shabila

**Affiliations:** 1 Maternity Teaching Hospital, Erbil, Kurdistan region Iraq; 2 Department of Obstetrics and Gynecology, College of Medicine, Hawler Medical University, Erbil, Kurdistan region Iraq; 3 Department of Community Medicine, College of Medicine, Hawler Medical University, Erbil, Kurdistan region Iraq

**Keywords:** Congenital anomalies, Neonate, Pattern, Associated factors

## Abstract

**Background:**

Congenital anomalies are a worldwide problem, causing perinatal and infant deaths and postnatal physical disabilities. This study aimed to determine the pattern and associated factors of the congenital anomalies in newborns delivered at the Maternity Teaching Hospital, Erbil city.

**Methods:**

All the births occurring in the labor room of the Maternity Teaching Hospital in Erbil city, Kurdistan region, Iraq between 1st April 2015 and the end of March 2016 were recorded. All babies with congenital anomalies were identified. The rate and common types of congenital anomalies were estimated. A case-control study was conducted involving all women who had babies with congenital anomalies and the same number whose babies had no congenital anomalies. Data were collected using a structured questionnaire.

**Results:**

Of the 35,803 recorded births in the Maternity Teaching Hospital, Erbil, 130 women delivered babies with at least one congenital anomaly, giving a rate of 3.63/1000 deliveries. The most common area for anomalies was the central nervous system (37.7%) followed by the musculoskeletal (23.1%) and gastrointestinal systems (20.8%). There was a statistically significant association between having a child with congenital anomalies and a maternal history of previous congenital anomalies (odds ratio [OR] 59.0, 95% CI 5.74–607.0), parental consanguinity (OR 6.26, 95% CI 2.42–16.19), and history of medical disorders (OR 153.2, 95% CI 25.9–905.4). Maternal occupation and smoking did not have any influence to develop congenital anomalies (OR 0.69, 95% CI 0.12–3.97 and OR 1.22,95% CI 0.19–7.93).

**Conclusion:**

Anomalies were most likely to be in the central nervous system. Maternal history of previous congenital anomalies, parental consanguinity, and history of medical disorders were the most important factors associated with congenital anomalies. This study provides baseline information for future prevention and better management of patients likely to have babies with congenital anomalies. More research is required to identify the factors responsible for the different types of congenital anomalies.

**Electronic supplementary material:**

The online version of this article (10.1186/s12884-018-2141-2) contains supplementary material, which is available to authorized users.

## Background

Congenital anomalies, also known as birth defects, include structural or functional anomalies of prenatal origin, resulting from an abnormality or defect that occurs during the development process [1]. Congenital anomalies accounted for 510,400 deaths worldwide in 2010, according to the Global Burden of Disease Study [2]. The prevalence of congenital anomalies at birth varies greatly worldwide, ranging from 1.07% in Japan to 4.3% in Taiwan [3]. Such a high variation in prevalence could be related to social, racial, ecological, and economic influences [3, 4].

The prevalence rates of congenital anomalies recorded in developing countries are likely to be underestimated due to unavailability of diagnostic capabilities or accurate medical records, as well as underreporting [5].

Congenital anomalies are broadly classified into either single-system or multiple-system malformations. The first type affects a single organ system or body part, and the second affects more than one organ system or body part [6–8]. Major congenital anomalies are defined as those that, if uncorrected, could result in considerable impairment of the normal body functions or even reducing the life expectancy. Minor congenital anomalies include the anomalies that cause no disability or have no significant physical or functional effects and can be regarded as normal variants [6, 9, 10].

Fetal anomaly scanning is the most effective method of reducing the prevalence of serious congenital abnormalities and increasing the survival rate of those born with these issues [11]. The finding of a correctable abnormality can serve to indicate that delivery should take place in a center with pediatric surgery facilities, and the discovery of a severe uncorrectable abnormality might result in offering pregnancy termination [11]. Survivors of congenital anomalies might have lifelong physical, mental, visual, and auditory disabilities if not managed appropriately [12], which can negatively affect the human and economic life of the person concerned, as well as their families and communities. Parental emotional responses, such as denial, feelings of guilt, worry, grief, and shame, occur after the birth of an infant with major anomalies. This response occurs even if the anomalies were diagnosed antenatally, highlighting the significance of proper counseling [13].

Developing population-based registries for the epidemiological surveillance of congenital anomalies is very important to unify records and provide better information about the distribution of common and rare types of the anomalies. The International Clearinghouse for Birth Defects Surveillance and Research is a voluntary non-profit international organization officially linked to the World Health Organization. It brings together congenital anomalies surveillance and research programs from all around the world, to investigate and prevent congenital anomalies and reduce their effect [14]. Neither it nor any other international birth defects surveys operate in our locality. Also, no national organizations that record congenital anomalies exist.

Knowledge about the prevalence of congenital anomalies is useful to obtain baseline rates, documenting changes over time, and identifying clues to the etiology of conditions. This knowledge is also helpful to plan and assess antenatal screening for congenital anomalies, especially for high-risk populations [15]. To the best of our knowledge, no study has been conducted in the Maternity Teaching Hospital of Erbil, Iraq, to determine the pattern of congenital anomalies and the associated risk factors. This study, therefore, aimed to determine the pattern of congenital anomalies in that hospital and assess the association between congenital anomalies and maternal characteristics.

## Methods

### Study design and setting

The study was conducted in the Maternity Teaching Hospital in Erbil city. The Maternity Teaching Hospital is the only public hospital in Erbil city, which provides delivery services. While most deliveries in Erbil city occur in the Maternity Teaching Hospital, deliveries also occur in homes and a number of smaller private hospitals. As we did not have access to the data about deliveries occurring in homes and private hospitals, we only included the deliveries that occurred at the Maternity Teaching Hospital in this study. Of all the 35,803 births that have occurred in the labor room of the Maternity Teaching Hospital in the city of Erbil city, in Kurdistan, Iraq between 1st April 2015 and the end of March 2016, any babies with congenital anomalies, including still-births and terminations, were identified. A total of 130 (0.36%) cases of congenital anomalies were recorded during the study period. Due to the lack of data about the maternal characteristics of all the 35,803 births and the logistical difficulties of collecting such data, it was decided to conduct a case-control study involving the 130 women who had babies with congenital anomalies during the study period and a similar number of women whose babies had no congenital anomalies.

### Study participants and sampling procedure

All the women who delivered babies with congenital anomalies in the Maternity Teaching Hospital within the study period were included in the study. The same number of women who had delivered babies with no congenital anomalies were also recruited as a control group.

The main inclusion criteria for selecting the controls was that they delivered a baby with no identified congenital anomaly on the same day as one of the women in the study group. Women of any age and any parity were included, and the delivery method included spontaneous vaginal delivery, pregnancy termination, induced labor, or cesarean section. We excluded the women who delivered before 24 weeks of gestation from the study.

All information about the women was recorded in a questionnaire designed for the study (Additional file 1) and completed by face-to-face interview after obtaining written informed consent. For the illiterate participants, the interviewer had read out the informed consent statement, and verbal consents were obtained from those who could not sign.

### Study variables

#### Dependent variable

The dependent variable was babies delivered with congenital anomalies in the Maternity Teaching Hospital in Erbil, Iraq, from 1st April 2015 to 31st March 2016.

#### Independent variables for cases and controls

The independent variables included maternal variables, particularly maternal age, parity, cigarette smoking during pregnancy, maternal diabetes mellitus and heart disease, number of antenatal clinic visits, classified as either adequate (more than four) or inadequate (less than four) in line with the World Health Organization guidance [16], consanguineous parents, previous history of congenital anomalies, and history of miscarriage. The educational level of the mother was classified into illiterate (i.e., cannot read or write), primary school, secondary school, or graduate from a college or institute.

### Study procedure

All mothers of babies with congenital anomalies were included, including cases referred to the termination of pregnancy committee in the hospital, and women who delivered babies whose anomalies were discovered by the obstetrician on call or the pediatrician during resuscitation. Other women giving birth in the hospital and delivering normal newborns were considered to be a control group for research purposes.

The diagnosis of congenital anomalies was in two ways. In the first group, the diagnosed was made by routine ultrasound during regular pregnancy follow-up and referred to the hospital’s termination of pregnancy committee. In the second group, women who delivered babies with congenital anomalies that were only discovered after birth, either by the obstetrician on call or the pediatrician during resuscitation. The researcher collected data from the women in both groups examined them and followed up the newborn during admission to the neonatal intensive care unit where detailed examinations, ultrasound, and x-rays were performed to reach a diagnosis. After confirming formal consent, the researcher conducted face-to-face interviews with the mothers. Data were collected about socio-demographic and clinical information, including maternal age, parity, parental consanguinity, history of diabetes mellitus, drug intake, history of congenital anomalies in the family, and the number of antenatal clinic visits. Mothers in both groups were followed up during and after delivery by the same researcher. The mode of delivery was recorded for all women in both groups. The birth weight of all babies was obtained using the same set of scales in the labor ward.

All newborns underwent a thorough physical examination (general and systemic examination), also performed by the researcher. A small number of infants died before assessment. A pediatrician or resident thoroughly assessed these infants in the pediatric department.

Echocardiography, x-ray, and cranial and abdominal ultrasonography were performed. Radiologists interpreted x-rays. Radiologists or a senior sonographer performed ultrasonography.

Using the International Statistical Classification of Diseases and Related Health Problems [17], we classified the patterns of congenital anomalies into:

1. Congenital malformation of the nervous system;
2. Congenital malformation of the musculoskeletal system;
3. Congenital malformation of the digestive system;
4. Congenital malformation of the circulatory system; and
5. Congenital malformation of the eye, ear, face, and neck.

### Statistical data analysis

The statistical package used for data management and analysis was SPSS version 17 (SPSS, Inc., Chicago, IL). Frequencies and percentages were calculated to describe the common types of congenital anomalies. The independent t-test and Chi-square tests were used to assess the differences in demographic characteristics of cases and controls. Univariate and bivariate regressions were performed where the congenital anomaly was the dependent variable, and maternal characteristics were independent variables.

Models were constructed in blocks: in the first, congenital malformation was forced in the model using enter method; in the next block, all potential confounders with *P* < 0.2 value in univariate analyses were introduced as candidate covariates using backward stepwise logistic regression method. Odds ratios (ORs) and 95% confidence intervals (CIs) were calculated to measure the strength and the significance of the association between congenital anomalies and maternal characteristics. The level of statistical significance (*P* value) was set at < 0.05.

## Results

Of the 35,803 births at the Erbil Maternity Teaching Hospital during the study period, a total of 130 (0.36%) cases of congenital anomalies were recorded. There was no statistically significant difference between cases and controls regarding the socio-demographic characteristics as shown in Table 1.

**Table 1:** Differences in the demographic characteristics of cases and controls

Variable		Study group (*n* = 130)	Controlled group (*n* = 130)	*P*
Age, years	Mean (SD)	27.5(6.9)	27.1(6.6)	0.628
Education	≤ secondary level	91(70.0)	93(71.5)	0.892
	> secondary level	39(30.0)	37(28.5)	
Occupation	Housewive	122(93.8)	115 (88.5)	0.189
	Employed	8(6.2)	15(11.5)	

The 130 cases of congenital anomalies included 103 (79.2%) live newborns and 27 (20.8%) stillbirths. Of the 103 live newborns, 81 (78.6%) needed admission to the neonatal care unit. In total, 20 (74.1%) of the 27 stillbirths were considered to be fresh and seven (25.9%) macerated. The highest proportion of congenital anomalies were those of the central nervous system (37.7%), followed by the musculoskeletal system (23.1%) and the gastrointestinal system (20.8%). Details of the distribution of congenital anomaly types are shown in Table 2. Of the 130 cases with congenital anomalies, 44 cases had multiple anomalies including 31 cases with multiple anomalies in different systems and 13 cases with multiple anomalies in the same system.

**Table 2:** Congenital anomalies distribution

Type of congenital anomaly	No.	%
Central nervous system	49	37.7
Musculoskeletal	30	23.1
Gastrointestinal tract	27	20.8
Eye, face, ear	3	2.3
Heart	7	5.4
Skin	4	3.1
Down syndrome	3	2.3
Conjoined twin	1	0.8
Unclassified	6	4.6
Total	130	100.0

The most common central nervous system anomalies were hydrocephalus (12.3%) and meningocele (12.3%), followed by anencephaly (11.5%) and spina bifida (2.3%). The most common musculoskeletal anomalies were clubfoot (7.7%), omphalocele (3.8%), and gastroschisis (3.1%). The most common gastrointestinal tract anomalies were cleft lip (18.5), cleft palate (16.2), Pierre Robin syndrome (4.6%) and esophageal atresia (3.8%) (Table 3).

**Table 3:** Distribution of the different congenital anomalies according to the body system

Central nervous system anomalies	No.	%
Hydrocephalus	16	12.3
Meningocele	16	12.3
Anencephaly	15	11.5
Spina bifida	3	2.3
Others^a^	4	3.1
Musculoskeletal anomalies		
Clubfoot	10	7.7
Omphalocele	5	3.8
Gastroschisis	4	3.1
Abnormal hand	3	2.3
Syndactyly	2	1.5
Polydactyly	2	1.5
Abnormal leg and pelvis	2	1.5
Others^b^	11	8.5
Gastrointestinal anomalies		
Cleft lip	24	18.5
Cleft lip	21	16.2
Pierre Robin syndrome	6	4.6
Esophageal atresia	5	3.8
Imperforate anus	3	2.3
Tongue tie	2	1.5

^a^Others included myelomeningocele, spinal dysmorphism, encephalocele, and microcephaly

^b^Others included oligodactyly, abnormality of the right hand, Amelia, amputated hand, amputated lower limb, fused toes and fingers, phocomelia, short leg, pigeon chest, scoliosis, and abnormal lower limb

According to the univariate and bivariate analysis, birth weight, maternal medical diseases, inadequate antenatal care, smoking, previous abortion, previous congenital anomalies, and consanguinity were significantly associated with congenital anomalies (Table 4).

**Table 4:** Association of maternal and neonatal factors with the risk of congenital anomalies

Variable		Total *N* (%)	Study group (*n* = 130)	Controlled group (*n* = 130)	OR	95% CI	*P*
Age, years	Mean (SD)	27.3(6.7)	27.5(6.9)	27.1(6.6)	1	0.97–1.04	0.628
Parity	Mean (SD)	1.9(1.7)	1.9(1.8)	1.9(1.7)	0.99	0.87–1.14	0.972
Birth weight, kg	Mean (SD)	2.97(0.5)	2.70(0.6)	3.24(0.4)	0.15	0.08–0.28	< 0.001
Education	≤ secondary level	184(70.8)	91(70.0)	93(71.5)	0.92	0.54–1.58	0.892
	> secondary level	76(29.2)	39(30.0)	37(28.5)	1	1	
Occupation	Housewive	237(91.2)	122(93.8)	115 (88.5)	1	1	0.189
	Employed	23(8.8)	8(6.2)	15(11.5)	0.5	0.20–1.23	
Medical diseases	Yes	71(27.3)	69(53.1)	2(1.5)	72.3	17.1–351.0	< 0.001
	No	189(72.7)	61(46.9)	128(98.5)	1	1	
Adequate antenatal care	Yes	155(59.6)	41(31.5)	114(87.7)	1	1	< 0.001
	No	105	89(68.5)	16(12.3)	15.4	8.14–29.35	
Smoking	Yes	21(8.1)	16 (12.3)	5 (3.8)	3.5	1.24–9.88	0.012
	No	239(91.9)	114(87.7)	125(96.2)	1	1	
Previous abortion	Yes	50(19.2)	14(10.8)	36(27.7)	0.31	0.16–0.61	0.001
	No	210(80.8)	116(89.2)	94(72.3)	1	1	
Previous congenital anomalies	Yes	28(10.8)	27(20.8)	1(0.8)	33.8	4.51–253.0	< 0.001
	No	232(89.2)	103(79.2)	129(99.2)	1	1	
Consanguinity	Yes	129(49.6)	90(69.2)	39(30.0)	5.25	3.09–8.90	< 0.001
	No	131(50.4)	40(30.8)	91(70.0)	1	1	
Sex of newborn	Male	149(57.3)	73(56.2)	76(58.5)	1	1	0.802
	Female	111(42.7)	57(43.8)	54(41.5)	1.09	0.67–1.79	

*CI* confidence interval, *OR* odds ratio

In logistic regression, there was no association between age, parity, education, occupation, smoking with congenital anomalies. Medical diseases (OR = 153.2,95% CI = 25.9–905.4), inadequate antenatal care (OR = 19.0,95% CI = 7.43–48.56), previous congenital anomalies (OR = 59.0,95% CI = 5.74–607.0), consanguinity (OR = 6.27,95% CI = 2.42–16.19) and low birth weight delivery (OR = 17.42,95% CI = 4.08–72.28) were associated with increased risk of congenital anomalies (Table 5).

**Table 5:** Logistic regression with factors associated with congenital anomalies

Variable		OR	95% CI	*P*
Occupation	Housewives	1	1	0.684
	Employers	0.69	0.12–3.97	
Medical diseases	Yes	153.2	25.9–905.4	< 0.001
	No	1	1	
Adequate antenatal care	Yes	1	1	< 0.001
	No	19	7.43–48.56	
Smoking	Yes	1.22	0.19–7.93	0.829
	No	1	1	
Previous abortion	Yes	0.12	0.02–0.66	0.014
	No	1	1	
Previous congenital anomalies	Yes	59	5.74–607.0	0.001
	No	1	1	
Consanguinity	Yes	6.27	2.42–16.19	< 0.001
	No	1	1	
Low birth	Yes	17.42	4.08–72.28	< 0.001
	No	1	1	

## Discussion

This study aimed to determine the pattern of congenital anomalies in the Maternity Teaching Hospital in Erbil city and assess the association between congenital anomalies and maternal characteristics. The patterns of congenital anomalies may differ over time or by geographical location, which may reflect complex interactions between environmental and genetic issues [18]. In this study, 3.63 congenital anomalies per 1000 births (0.36%) were recorded in the Maternity Teaching Hospital in Erbil. This result is probably an underestimate of the prevalence of congenital anomalies because the study was hospital-based and did not cover the whole Kurdistan region or even the city of Erbil. It is also significantly lower than the lowest estimated rate from elsewhere, 1.07% in Japan [3]. No population-based registry of congenital anomalies exists in Iraq, which may lead to underestimation. It is, however, thought likely that most pregnancies involving congenital anomalies in the city were managed in this hospital, because it is the only government tertiary hospital in Erbil.

The recorded rate of major congenital anomalies in Turkey was 2.9/1000 births between 2000 and 2004 [19]. It was 7.76 per 1000 births in Basrah, Iraq in 1998 [20], and 12.36 per 1000 births in Baghdad in 2007 [21]. The rate of congenital anomalies reported in this study is also lower than previously reported in Iran (an average of 16.55 per 1000 total births, increasing from 10.46 in 2000 to 17.01 per 1000 births in 2004) [22]. These variations in the reported prevalence of congenital anomalies in locations that are geographically close together could be explained by social and racial influences, as these are commonly known to affect genetic disorders. Results in previous studies have also varied by the investigation’s background, type of sample studied, and observation period [23, 24].

The central nervous system anomalies, which occurred both as single-system and multiple-system malformations, were the most common type of anomaly found in this study. This finding is similar to that reported in the Cross Rivers State in the South of Nigeria [25], and Jos in the North Central region of Nigeria [26]. The high occurrence of central nervous system anomalies could be explained by a lack of foods fortified with folic acid, very low conventional intake of folic acid, and inadequate dietary intake of foods rich in folic acid, such as vegetables. These folic acid intake problems were probably due to poor appetite and nausea during pregnancy, and poor antenatal care. Better antenatal care might have allowed diagnosis of these anomalies at an earlier stage. Studies have shown that daily maternal intake of folic acid alone or in the form of multivitamin supplements before conception until the first trimester of pregnancy can help in preventing the occurrence and recurrence of neural tube defects [27, 28].

A significant association was observed between a familial history of congenital anomalies and the risk of new congenital anomalies in this study. Our observation is consistent with the findings of a previous report that occurrence of a congenital anomaly linked to a chromosomal abnormality, whether live-born or stillborn, increased the risk of chromosomal defects in future pregnancies [29]. Couples with one child with a neural tube defect but no additional family history have a recurrence rate of 2–5%, and couples with one child with Down syndrome have a recurrence risk of 1% [16]. These findings are consistent with other studies in Egypt, which observed a significant association between a family history of congenital anomalies and their occurrence. The association was found to be even stronger among siblings from consanguineous marriages [29–31]. Consanguineous marriages are believed to have an important contribution to the risk of congenital abnormalities [1, 32]. The risk of a child having a recessively inherited condition is higher among related parents, and this risk increases with the closeness of the relationship [1, 33].

In this study, there was a statistically significant association between having medical diseases in the mothers, primarily diabetes mellitus and delivering babies with congenital anomalies. Prior research has established clear links between maternal glycemic control and the risk of developing congenital anomalies [31, 34, 35]. In another study conducted in Egypt on live-born babies, the incidence of minor congenital anomalies among infants of diabetic mothers was 18%, while the incidence was 11% for the major congenital anomalies. The later was 4.6 times higher than in the general population [36].

In this study, 12.3% of mothers in the study group were cigarette smokers, compared with 3.8% of controls, including both actual and passive smokers. However, we could not detect a statistically significant association between smoking and deliver babies with congenital anomalies. Other studies had revealed that maternal smoking between one month before conception to the third month of pregnancy, was associated with congenital anomalies of the heart, cleft lip and sometimes cleft palate. A weaker association was found with cleft palate alone. The association with smoking is more evident in mothers not taking folic acid supplements. However, there was no association between cleft lip or cleft palate alone and passive exposure to smoking [37]. Exposure to carbon monoxide and/or nicotine from smoking might be primarily responsible for these defects. The effect of carbon monoxide can be due to diminishing the oxygen supply to the body’s tissues. Nicotine might act through enhancing the release of hormones responsible for constricting vessels and reducing the blood supply to the placenta and uterus. These factors result in having diminished oxygen and inadequate nutrients reaching the fetus and thus increasing the risk of congenital anomalies [31].

Congenital anomalies in this study were significantly associated with having inadequate antenatal care. With adequate antenatal care, pregnant women are often provided with health education on various issues such as the importance of proper nutrition, how to avoid teratogens, and prevention of maternal infections. Also, folic acid supplements are often offered during antenatal care visits [5]. Similar findings have been observed in Brazil, where few (no more than four) or no prenatal clinic visits were significantly associated with the occurrence of congenital anomalies [38].

## Limitations

This study had some limitations. One was that the study sought to detect mostly external (overt) congenital anomalies among neonates within the first 48 h of birth, relying only on clinical examinations to make a diagnosis. Neither cytogenetic analysis nor autopsies for stillbirths were performed, because these procedures are expensive and have limited availability in our locality. It is therefore likely that the study missed some congenital anomalies that do not present early in life, such as heart defects, pyloric stenosis, and anomalies of the urinary system, which could also explain the low level of defects found compared with other studies.

Despite the limitations, this study has several strengths. One is that it was conducted in the only public hospital in the city of Erbil, which records more than 30,000 deliveries annually. Most cases in the city with congenital anomalies are referred to this hospital, which has a termination of pregnancy committee responsible for advising the pregnant woman about the type of anomaly of their fetus and if it is compatible with life or not. Each pregnant woman with a malformed fetus terminated via cesarean section or induction of labor was formally seen by the committee. This research, to the best of our knowledge, is the first to be conducted on the population of Kurdish women living in the Kurdistan region in Iraq. Another study strength is that the data came from live births and stillbirths (both macerated and fresh), which increases the reporting accuracy of the prevalence of congenital anomalies. No data were lost or missing, and all women recruited into the study agreed to participate in it.

This study did not provide direct benefits to study participants, but we recommend that future studies should provide information to both help women prevent congenital anomalies and better management of patients with congenital anomalies. More research is required to identify possible determinants responsible for the various types of congenital anomalies, and we recommend future studies to evaluate specific groups of congenital anomalies, including their risk factors and prevalence rate.

## Conclusion

Anomalies were most likely to be in the central nervous system. Maternal history of previous congenital anomalies, parental consanguinity, and history of medical disorders were significantly associated with an increased risk of congenital anomalies. Adequate antenatal care and previous abortions were significantly associated with a decreased risk. No statistically significant associations were found for other maternal and neonatal factors. This study provides useful information on the magnitude and spectrum of congenital anomalies diagnosed soon after birth among neonates at the Maternity Teaching Hospital in Erbil, in the Kurdistan region of Iraq.

## Additional file

Additional file 1: Structured questionnaire for assessing the pattern of congenital anomalies at birth and their correlations with maternal characteristics in the Maternity Teaching Hospital, Erbil city, Iraq. (DOCX 14 kb)

## Abbreviations

CI: Confidence Interval.
OR: Odds ratio
