# Supplementary material for: Pattern of congenital anomalies at birth and their correlations with maternal characteristics in the maternity teaching hospital, Erbil city, Iraq
Source: BMC Pregnancy Childbirth. 2018 Dec 18;18:501. doi: 10.1186/s12884-018-2141-2 (PMC6299654; doi:10.1186/s12884-018-2141-2)
Supplement: Supplementary file 1 — Structured questionnaire for assessing the pattern of congenital anomalies at birth and their correlations with maternal characteristics in the Maternity Teaching Hospital, Erbil city, Iraq. (DOCX 14 kb) [file 12884_2018_2141_MOESM1_ESM.docx]

**Additional file 1** Pattern of congenital anomalies at birth and their correlations with maternal characteristics in the Maternity Teaching Hospital, Erbil city, Iraq

***Serial no.:***

**Information about the woman:**

**Date of birth: / /20**

**Name: Age:**

**Address: Tel no.:**

**Level of education:** Illiterate Primary school Secondary school College/institute

**Occupational status:** Housewife Employed

**Parity:** **Previous abortion:** Yes No

**Previous congenital anomalies:** Yes No ***Type*:**

**Consanguinity** Yes No ***Degree:***

**Any medical disease:** Yes No ***Type:***

**Smoking:** Yes No

**Drug used during pregnancy:** Yes No ***Type of drug:***

***At which trimester*: 1^st^ 2^nd^ 3^rd^**

**Adequate antenatal care:** Yes No

**Gestational age: weeks**

**Pregnancy:** Single Twin Triple

**Mode of delivery:** Normal vaginal Cesarean section

**Fetal outcome:**

**Birth weight:** kg

**Fetal status:** Alive Dead

**Sex:** Male Female

**Congenital anomalies:** Yes No

**If yes, types and details of the anomalies:**
